# 3D Image-Guided Percutaneous Radiofrequency Thermocoagulation of the Maxillary Branch of the Trigeminal Nerve Through Foramen Rotundum for the Treatment of Trigeminal Neuralgia

**DOI:** 10.1097/MD.0000000000001954

**Published:** 2015-11-13

**Authors:** Tongqing Xue, Weixi Yang, Yunhu Guo, Weiwei Yuan, Jianhu Dai, Zhenxin Zhao

**Affiliations:** From the Department of Interventional Radiology, The First Affiliated Hospital of Soochow University (TX); Department of Pain and Interventional Radiology (TX, YG, WY, JD, ZZ), Huaiyin Hospital of Huai’an City; and Department of Burn and Plastic Surgery (WY), Huai’an First People's Hospital, Nanjing Medical University, Huai’an, China.

## Abstract

Percutaneous radiofrequency thermocoagulation of the trigeminal ganglion through the foramen ovale is a well-established procedure for the treatment of trigeminal neuralgia (TN). However, this approach can be tricky when individual trigeminal sub-branch nerve block is required. We report our initial experience of image-guided radiofrequency thermocoagulation of the maxillary branch through the use of foramen rotundum.

From February 2012 to February 2015, we treated 25 patients with isolated TN of the maxillary branch. Radiofrequency thermocoagulation of the maxillary branch through the foramen rotundum was performed under fluoroscopy. TN pain was evaluated using the visual analogue scale both before and after the procedure.

The mean preoperative visual analogue scale score was 8.6 ± 0.8. The pain completely disappeared after the initial procedure in 22 patients and after a second procedure in 2 patients. An additional patient had a postoperative visual analogue scale score of 2 and did not undergo further treatment. Facial numbness occurred in 23 patients but was tolerable. Patients were followed up for a mean of 14.74 months (range, 1–29 months). Recurrence was observed in 9 patients (36%) during the follow-up period. All recurrences were well managed with repeat procedures.

Percutaneous radiofrequency thermocoagulation of the maxillary branch through the foramen rotundum under fluoroscopy is a safe and effective procedure for the treatment of isolated TN of the maxillary branch.

## INTRODUCTION

Trigeminal neuralgia (TN) is a neuropathic disorder characterized by intense and distinctive pain in the distribution of one or more branches of the fifth cranial nerve. Diagnosis of TN is typically based solely on the patient's history and description of pain. Pain occurs in paroxysms, with each episode of pain lasting from a few seconds to several minutes with no pain experienced in between.^1^ Although the reported prevalence of TN is in the range of 4 to 5 per 100,000, a recent population-based study of over 3000 subjects in Germany found a lifetime prevalence of TN at around 0.3%, nearly 2 orders of magnitudes higher, at around 0.3%.^2–4^ Treatment options for TN are quite diverse, ranging from pharmacologic, radiosurgical, and other minimally invasive percutaneous techniques to major intracranial nerve exploration and decompression. Typically, patients with TN are first treated with medications, including carbamazepine or oxcarbazepine. If the pain cannot be controlled using conservative treatments, surgical interventions are then usually considered.^5^

Percutaneous thermocoagulation of the branches of the trigeminal nerve and the trigeminal ganglion has long been used in the treatment of TN. One possible mechanism for this technique involves differential thermocoagulation of trigeminal rootlets, whereby the action potentials of nociceptive fibers (A-δ and C) are blocked at lower temperatures than those that transmit tactile sensation.^5,6^ However, histologic animal studies have indicated that the induced injury affects all sizes of nerve fibers, regardless of their myelination status.^7^ Therefore, the success of the procedure may rely on reducing the overall sensory input to the trigeminal root. Radiofrequency thermocoagulation through the foramen ovale can be a difficult and time-consuming procedure for both the physician and the patient, requiring very precise placement of the needle near the Gasserian ganglion. Failure to position the needle properly can lead to treatment failure, recurrence, or potentially serious complications that include, but are not limited to, nonspecific block, intracranial hemorrhage, and infection.^8–10^

We present our initial experience with percutaneous thermocoagulation of the maxillary branch of the trigeminal nerve through the foramen rotundum, similar to that first described by Huang et al in 2014, under the 3D fluoroscopic guidance in a series of 25 cases.

## MATERIALS AND METHODS

### Patient Characteristics

From February 2012 to February 2015, 25 patients with isolated TN of the maxillary branch were treated with radiofrequency thermocoagulation through the foramen rotundum under fluoroscopic guidance at our hospital. Patients were all medically refractory and included 12 men and 13 women with a mean age of 64.4 ± 11.1 years (range: 26–82 years). The left side of the face was affected in 14 patients, whereas the right side was affected in 10, and 1 patient experienced bilateral symptoms. The disease course ranged from 1 month to 16 years (Table 1).

**TABLE 1:**
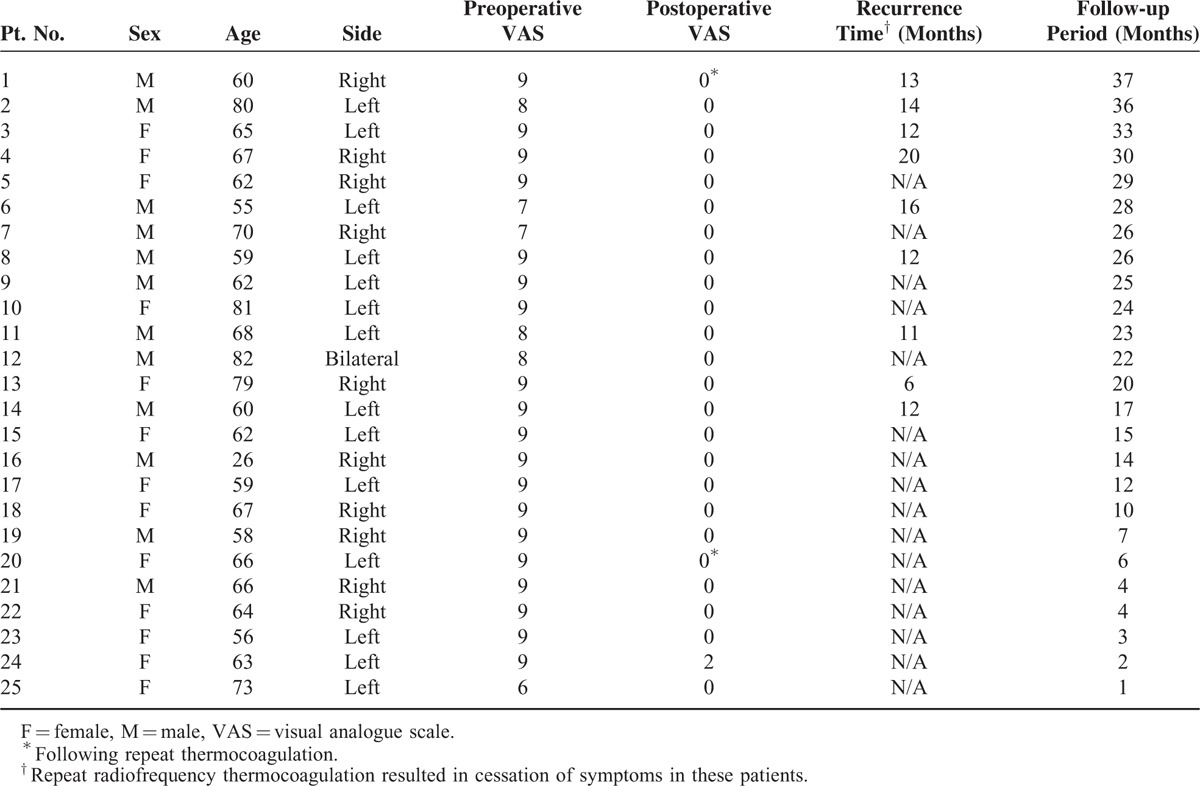
Patient Characteristics

Patients with symptoms of primary TN, pain in the distribution of the maxillary branch but not in the distribution of the ophthalmic or mandibular branches, complete disappearance of pain after a maxillary branch block, and/or a history of failed treatment(s) or recurrence of pain were included in this study. Patients with severe systemic disease, cardiac or pulmonary dysfunction, and/or secondary TN were excluded.

This study was approved by the Institutional Review Board of Huaiyin Hospital of Huai’an City. Each patient has signed a written informed consent form.

### Surgical Procedure

**FIGURE 1:**
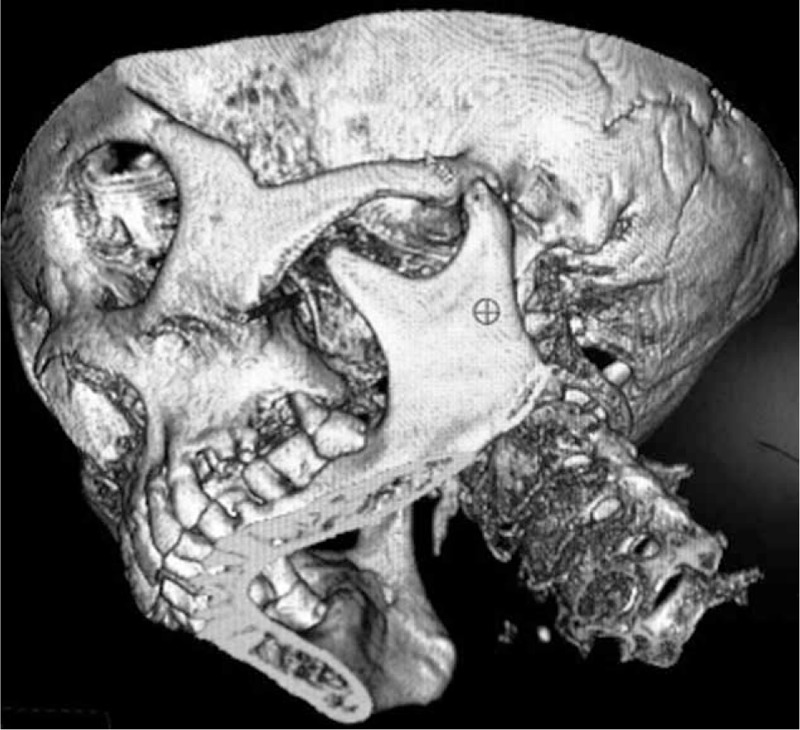
A 3D CT reconstruction showing the trajectory of the needle after insertion. CT = computed tomography.

**FIGURE 2:**
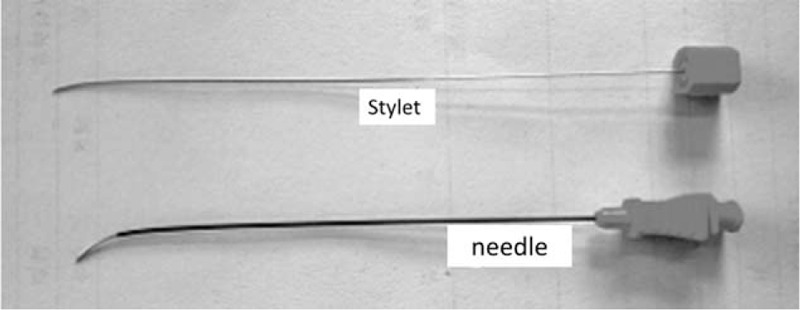
To facilitate needle positioning, a 21-gauge radiofrequency needle was bent at 1 cm from the needle tip to form a 40° curve.

Approximately 30 minutes before surgery, patients were administered 1 KU of hemocoagulase (Bang Ting, Aohong Pharmaceutical Co. Ltd., Jinzhou, China) and 5 mg of morphine subcutaneously. Patients were placed in the supine position on an angiography table of a biplane fixed x-ray system with 3D capability (Allura Xper FD20, Koninklijke Philips N.V., Amsterdam, Netherlands) with the head fixed in a slightly extended position. The C-arm was adjusted into an anteroposterior projection, with the detector positioned caudally toward the affected side. With this positioning, the projection of the ipsilateral pterygopalatine fossa was inferior to the ipsilateral zygoma, posterior to the maxillary bone, and anterior to the coronoid process of the mandible (Fig. 1). The entry point for the percutaneous treatment was the projection of the pterygopalatine fossa on the cheek. The entry point was anesthetized locally using 1 mL of 1% lidocaine. To facilitate needle positioning, a 21-gauge radiofrequency needle was bent at 1 cm from the needle tip to form a 40° curve (Fig. 2).

**FIGURE 3:**
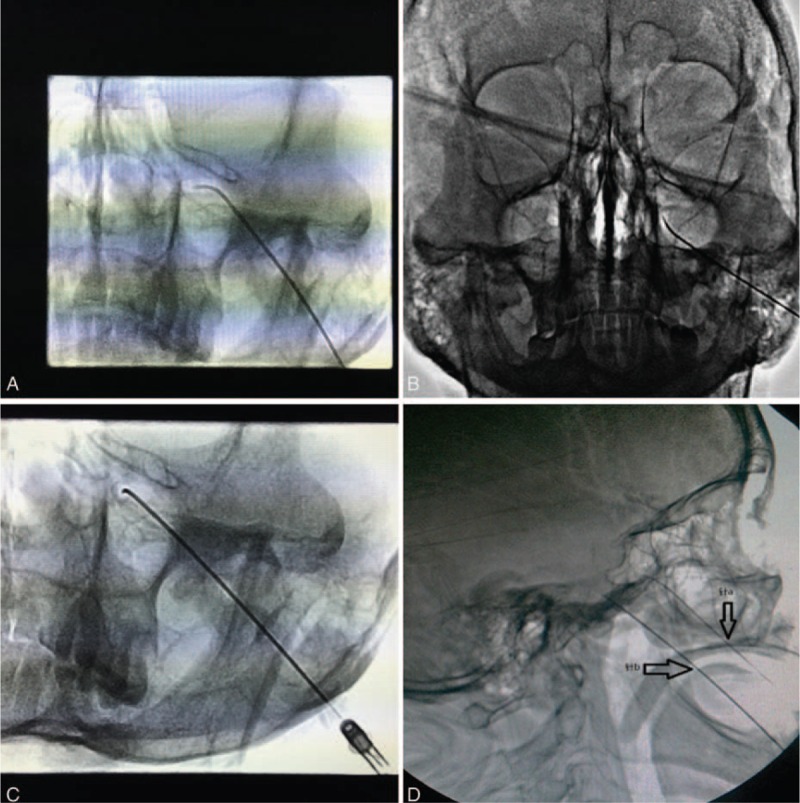
(A) Anteroposterior x-ray image showing the projection of the bilateral petrous pyramids onto the middle portion of the maxillary sinus. Foramen rotundum is found within the maxillary sinus superior to the petrous pyramid. (B) An anteroposterior view with the curved needle advanced through the posterolateral wall of the maxillary sinus toward foramen rotundum. (C) The needle was rotated in order for the tip to face posteriorly toward the foramen and advanced until through the foramen. (D) A lateral x-ray showing the difference between needle insertion at foramen ovale and foramen rotundum. The lower needle was advanced through foramen ovale using the Hartel method.

The C-arm was adjusted to project the bilateral petrous pyramids onto the middle part of the maxillary sinus, and foramen rotundum was identified within the maxillary sinus superior to the petrous pyramid (Fig. 3A). Using posteroanterior fluoroscopy, the needle and stylet were introduced into the posterolateral wall of the maxillary sinus and advanced toward foramen rotundum. The needle tip was positioned anteriorly to the pterygopalatine fossa and foramen rotundum (Fig. 3B). The needle was rotated so that the tip faced posteriorly toward foramen rotundum and advanced until the tip was within the foramen (Fig. 3C). The C-arm was placed in a lateral position and the needle was slowly advanced until the tip was observed entering the middle fossa (Fig. 3D). In some cases, patients experienced paresthesia along the course of the maxillary branch of the trigeminal nerve during advancement of the needle through the foramen rotundum to the middle cranial fossa. In 15 of these cases (60%), patients could not tolerate this paresthesia and the needle was withdrawn to the external opening of the foramen rotundum so that 1 mL of 2% lidocaine could be injected before re-advancing the needle.

**FIGURE 4:**
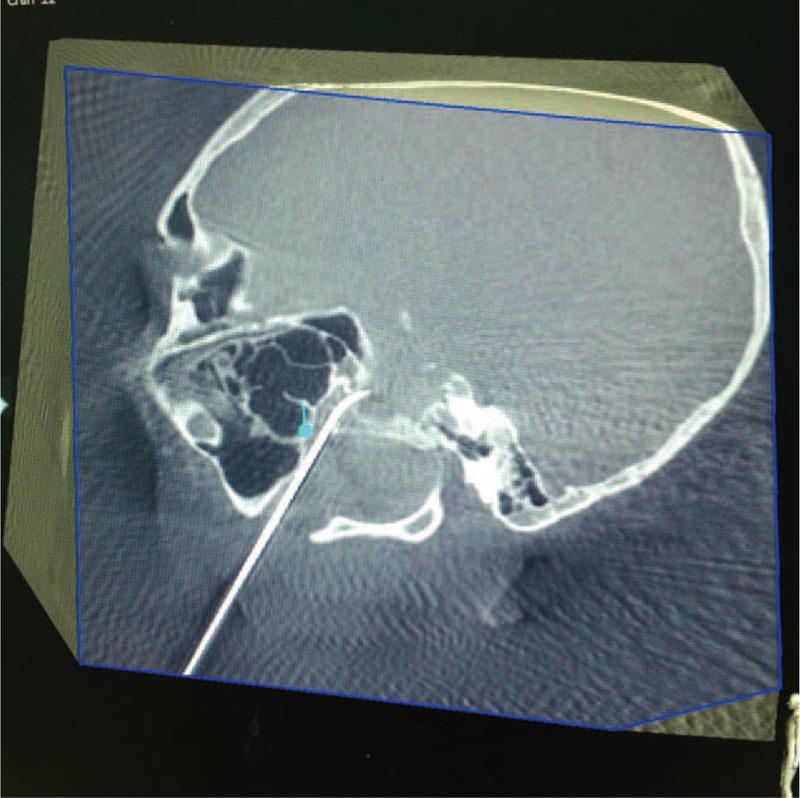
A lateral CT image showing the position of the needle in foramen rotundum. CT = computed tomography.

The stylet was removed from the needle and a radiofrequency electrode (ET-20S, Smith & Nephew, USA) was inserted through the needle. The probe was stimulated with an initial pulse of 50 Hz. Patients experienced paresthesia in the distribution area of the maxillary branch at 0.2 V which intensified with increased voltage. No paresthesia was noticed in the distribution areas of the ophthalmic and mandibular branches at 0.7 V, indicating that the needle was correctly placed within the maxillary branch and not within the ophthalmic and mandibular branches. The position of the needle within the foramen was confirmed using 3D-reconstruction in the coronal, sagittal, and needle trajectory planes (Fig. 4).

After positioning of the needle was confirmed, the radiofrequency probe was removed, 0.3 mL of 2% lidocaine was slowly injected through the needle, and patients were monitored for 3 to 10 minutes until the pain along the maxillary branch was replaced by a feeling of numbness. The radiofrequency electrode was reinserted into the needle and thermocoagulation was performed at 75°C for 4 minutes. During thermocoagulation, functions of the ophthalmic and mandibular branches were repeatedly assessed by physical examination and patient feedback.

### Assessment of Pain Reduction

The visual analogue scale (VAS), a widely used metric for measuring pain, was completed by patients both pre- and postoperatively.^11^ Patients were shown a horizontal line marked from 0 (no pain) to 10 (most severe pain) and asked to rank the severity of their pain.

## RESULTS

The mean preoperative VAS score was 8.6 ± 0.8. Of the 25 patients, 22 (88%) indicated a postoperative VAS scores of 0, indicating complete resolution of pain after the procedure (Table 1). Of the remaining 3 patients, 2 had postoperative VAS scores of 5 and 3, respectively, and each subsequently underwent a second interventional procedure that resulted in complete resolution of pain. The remaining patient had a postoperative VAS score of 2 which resolved several weeks postoperatively without subsequent treatment. Mild persistent facial numbness was noted in 23 patients and no other complications were observed. Patients were followed up between 1 and 29 months, with a mean follow-up period of 14.74 ± 11.34 months. Recurrence was observed in 9 patients (36%) at follow-up. All patients with recurrent symptoms were successfully managed with repeat thermocoagulation, as of the time of publication. Thus, the total reoperation rate was 44%.

Operative time was very short at an average of 15 minutes. Average puncture time (from insertion to placement) was about 5 minutes with an average radiofrequency time of approximately 4 minutes. Surgical complexity was similar to that of traditional percutaneous image-guided trigeminal thermocoagulation.

## DISCUSSION

Trigeminal neuralgia is a neuropathy involving intense pain limited to the distribution of 1 or more of the branches of the trigeminal nerve.^1^ Percutaneous treatment modalities for TN consist of balloon compression, glycerol rhizotomy, and radiofrequency thermocoagulation. All 3 treatments have been shown to induce pain relief by directed injury to the trigeminal nerve and are generally considered to be safe, efficient, and effective.^12^ Recent improvements in percutaneous radiofrequency thermocoagulation have involved improving the targeting accuracy of the procedure through the use of intraoperative computed tomography neuronavigation and frameless stereotactic cannulation of the foramen ovale, with good success.^13–15^

Xu and colleagues^16^ described their experience with 54 patients and reported increased rates of long-term pain relief (85% vs 54% at 12 months and 62% vs 35% at 36 months) in the patients undergoing percutaneous radiofrequency thermocoagulation with navigation compared with the previously standard procedure. A later study by Yang and colleagues^17^ in 2010 involved 79 patients with TN who underwent radiofrequency rhizotomy. Yang et al reported no difference in surgical outcome or pain relief between CT-guidance with 3D reconstruction and fluoroscopy-guided groups. However, CT-guidance significantly decreased the median time for needle placement (14 vs 40 min) and intraoperative patient discomfort.

The classic approach to radiofrequency thermocoagulation involves placement of the needle through foramen ovale. However, tracing the maxillary branch of the trigeminal nerve from its origin at the Gasserian ganglion involves passing through foramen rotundum to exit the cranium, subsequently crossing the pterygopalatine fossa, and entering the orbit through the inferior orbital fissure. Thus, an approach through the foramen rotundum, rather than the foramen ovale, has the potential to simplify targeted treatment of the maxillary branch of the trigeminal nerve in TN patients.

Huang et al proposed a computed tomography-guided technique through foramen rotundum to block the maxillary branch of the trigeminal nerve^4^ and found that, compared to an approach through foramen ovale, thermocoagulation through foramen rotundum showed similar results for pain relief both immediately postoperative and at 1-year follow-up. In addition, approaching the trigeminal nerve through the foramen rotundum was associated with a shorter procedural time and fewer serious adverse effects including masticatory weakness and corneal perforation. All patients treated by Huang et al through foramen rotundum experienced some degree of facial numbness. However, while this group had a greater incidence of facial numbness than the foramen ovale group, their degree of numbness was typically less extensive.

In the present study, we performed a similar technique using 3D image-guided radiofrequency thermocoagulation of the maxillary branch through the foramen rotundum. No patient underwent more than 2 intervention procedures and all but 1 patient experienced immediate and sustained pain relief after initial or secondary treatment. Recurrence occurred in a minority of patients (36%), similar to that of comparable techniques. The complications observed following this technique were mild and usually did not require additional treatment. No incidence of masticatory weakness or corneal perforation—both known complications of radiofrequency coagulation through foramen ovale^4^—were observed. The vast majority of our patients (92%) experienced facial numbness; however, this numbness was considered tolerable and corroborates the findings by Huang et al that numbness caused by a foramen rotundum approach is typically not extensive or deteriorative to quality of life.

## CONCLUSION

This series of 25 cases of TN of the maxillary nerve treated with 3D image-guided radiofrequency thermocoagulation through foramen rotundum demonstrates that this technique may help mitigate the risk of iatrogenic damage to the ophthalmic and mandibular nerves through repeated movements of the needle intracranially—which could result in weakened mastication and/or corneal damage. Furthermore, this technique may reduce the need for repeated intraoperative electrical stimulation and thus reduce patient discomfort. However, larger prospective clinical studies are necessary to determine the true efficacy of at this technique.
